# Benefit of continuous positive airway pressure on optic nerve damage in patients with obstructive sleep apnea

**DOI:** 10.1007/s11325-025-03336-w

**Published:** 2025-04-30

**Authors:** Teresa Diaz de Terán, Ignacio Boira, Andrea Cerveró, Alfonso Casado, Alicia Lopez-de-Eguileta, Soraya Fonseca, Pedro Muñoz, Claudia Nebot, Antonello Nicolini, Paolo Banfi, Paolo Solidoro, Mónica González

**Affiliations:** 1 Department of Pneumology, Sleep and Non-Invasive Ventilation Unit, ’Marqués de Valdecilla’ University Hospital, Santander, Spain; 2 University of Cantabria, Institute for Research ’Marqués de Valdecilla’ (IDIVAL), Santander, Spain; 3 Department of Pneumology, Sleep and Non-Invasive Ventilation Unit, University Hospital of San Juan Alicante, Alicante, Spain; 4 Department of Ophthalmology, ’Marqués de Valdecilla’ University Hospital, University of Cantabria, Institute for Research ’Marqués de Valdecilla’ (IDIVAL), Santander, Spain; 5 Primary Care Management of Cantabria, University of Cantabria, Institute for Research ’Marqués de Valdecilla’ (IDIVAL), Santander, Spain; 6 IRCCS Fondazione Don Carlo Gnocchi, Milan, Italy; 7 AOU Città della Salute e della Scienza di Torino, Torino, Italy; 8 Department of Pneumology, Sleep and Non-Invasive Ventilation Unit, University Hospital, University of Cantabria, Institute for Research ’Marqués de Valdecilla’ (IDIVAL), Av. Valdecilla, 25, Santander, Cantabria, 39008 Spain

**Keywords:** Obstructive sleep apnea, Hypoxic burden, Retinal damage, BMO-MRW, RNFL

## Abstract

**Purpose:**

The purpose of our study was to evaluate the effectiveness of CPAP in increasing the thickness of retinal layers. Other aims were to assess retinal and optic nerve damage predictors in OSA and establish predictors of poor response to CPAP treatment in optic nerve damage.

**Methods:**

A prospective cohort study with consecutive inclusion of the first 3 patients who attended for treatment each day. All patients underwent a diagnostic polygraph, and patients with moderate-severe OSA treated with CPAP were recruited. Optical Coherence Tomography (OCT) was performed within 3 days of the patient’s inclusion and 12 months after the start of CPAP treatment.

**Results:**

Data from 37 patients with OSA were analysed. After 12 months of CPAP treatment, there was a significant improvement in the thickness of the superotemporal Bruch’s membrane opening-minimum rim width (BMO-MRW) (316.54 to 318.23 μm, p-value = 0.08). There was a non-significant improvement in the thickness of nasal, inferonasal and superonasal retinal nerve fibre layers. In a multivariate analysis, HB and Type 2 diabetes mellitus have been associated with an increased odds ratio (OR) of retinal and optical nerve damage (OR = 3.58, *p* = 0.03 and OR = 4.344, *p* = 0.042, respectively).

**Conclusion:**

BMO-MRW thickness may assess early damage induced by OSA and the response to CPAP. HB is a predictor of retinal and optic nerve damage in patients with OSA. CPAP treatment has a long-term protective effect on the retina and optic nerve.

## Introduction

Obstructive sleep apnea syndrome (OSA) is a sleep and respiratory disorder characterised by intermittent complete (apnea) or partial (hypopnea) upper airway obstruction during sleep due to the collapse of upper airway^1^. Repeated apnea/hypopnea episodes induce significant hypoxemia leading to the production of inflammation, reactive oxygen species, activation of the sympathetic nervous system and vascular endothelial damage [1, 2].

It is estimated that the prevalence of OSA is around 4% in men aged 30–60 years and 2% in women aged 30–60 years in western countries [3]. In Spain, the prevalence between the ages of 30 and 70 years is estimated at 19% in men and 15% in women [4]. Apnea-Hypopnea Index (AHI) is the measure most commonly used to diagnose OSA, but it has many limitations due to poor correlation with symptoms and nocturnal desaturation. The Hypoxic Burden (HB) measurement has recently been introduced. It represents the total area under the oxygen saturation curve from a pre-event baseline desaturation and assesses the frequency, depth and duration of desaturation related to the respiratory event. HB has been associated with major cardiovascular events, heart failure, arterial hypertension, stroke and chronic renal failure [5]. Continuous positive airway pressure (CPAP) is the first-line treatment for moderate to severe OSA and is essential in improving patients’ symptoms and in decreasing the metabolic and vascular consequences [6].

Several neuro-ophthalmological diseases have been related with OSA. The association between OSA and glaucoma has been of great interest because of the high prevalence of both diseases. Several meta-analyses have evidenced a correlation between the two entities and a lower thickness of the retinal nerve fibre layer (RNFL) in patients with OSA [7].

Treatment with CPAP in glaucoma can increase intraocular pressure but may also improve optic nerve perfusion and decrease disease progression. There is only one study that evaluates retinal thickness after CPAP treatment, and this shows favourable short-term results [8].

Another ophthalmologic disease with a link to OSA is Non-Arteritic Anterior Ischemic Optic Neuropathy (NA-AION). Patients with OSA have a 6-fold increased risk of NA-AION mainly due to dysfunction of blood flow regulation of the posterior ciliary arteries which supply the optic nerve [9]. A retrospective study of 2 million patients found that patients with untreated OSA had an increased risk of NA-AION compared to patients treated with CPAP [10].

Papilledema has also been associated with OSA due to increased retinal vascular pressure, cerebral vasodilatation, rising cerebral blood flow and reduced venous return to the central nervous system (ICP) [11]. A clinical trial showed an improvement in papilledema in patients with OSA who had improved disease control [12].

In central serous chorioretinopathy (CSC), there are several physiological mechanisms that link both entities [9, 13] In a meta-analysis patients with CSC have a significantly higher risk of OSA than the controls [14]. Other chorioretinal pathologies associated with OSA are diabetic retinopathy and age-related macular degeneration (ARMD) with a poorer response to anti-VEGF [15].

In terms of retinal vascular changes produced in OSA, increased vascular tortuosity, decreased arteriovenous radius, narrowing of the retinal arteriole, and a reduced venous and arterial pulse have been described. Furthermore, the proinflammatory state of OSA increases the risk of retinal vein obstruction [16].

**Fig. 1:**
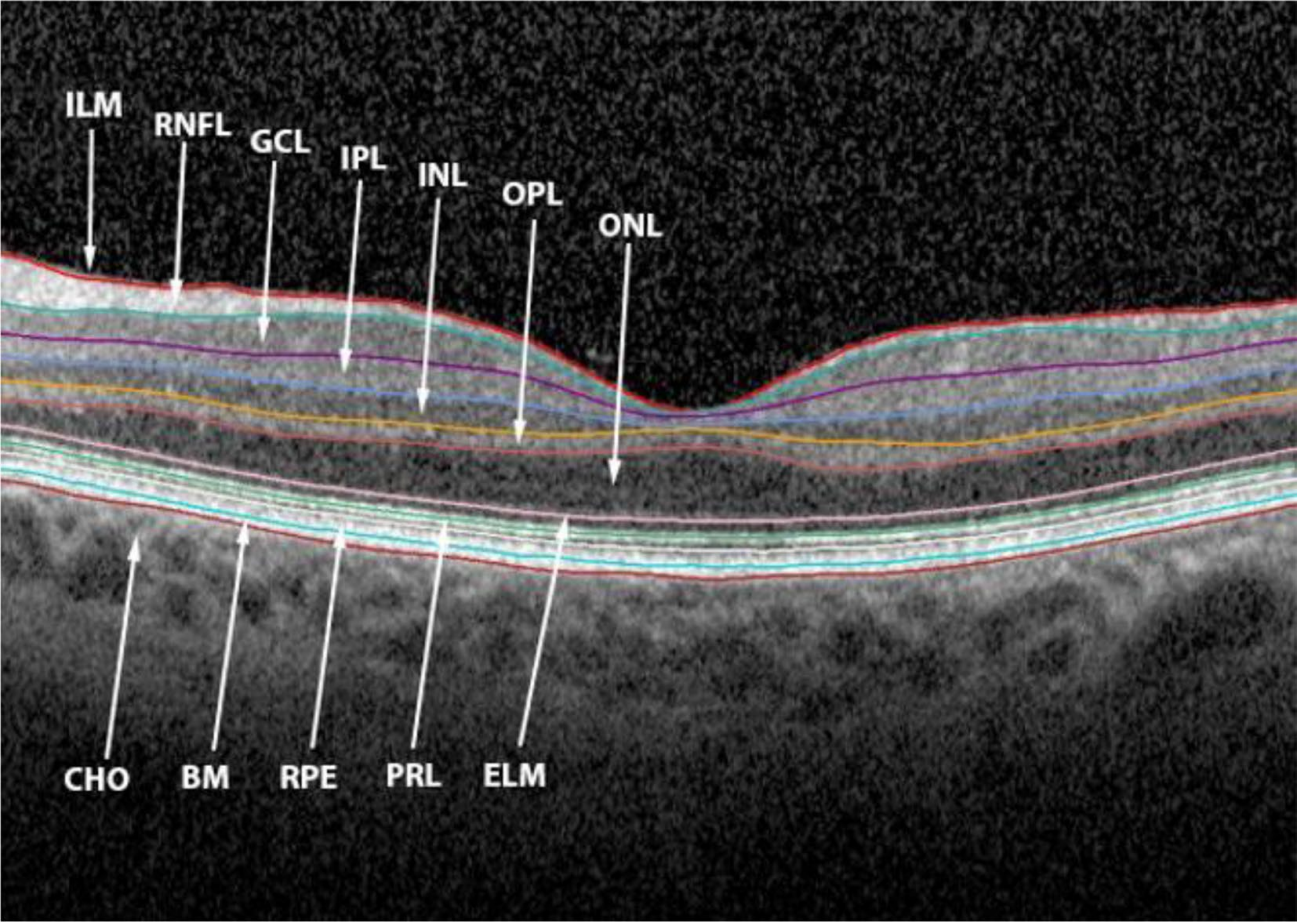
Retinal layers in optical coherence tomography (OCT). Inner Limiting Membrane (ILM), Retinal Nerve Fibre Layer (RNFL), Ganglion Cell Layer (GCL), Inner Plexiform Layer (IPL), Inner Nuclear Layer (INL), Outer Plexiform Layer (OPL), Outer Nuclear Layer (ONL), External Limiting Membrane (ELM), Photoreceptor Layer (PRL), Retinal Pigment Epithelium (RPE), Bruch’s Membrane (BM), Choroid (CH)

The retina consists of several layers, including the retinal pigment epithelium (RPE), which absorbs light and supports photoreceptor cells. The photoreceptor layer (PRL) comprises rods and cones, and converts light into electrical signals for vision. The outer nuclear layer (ONL) houses photoreceptor cell bodies, while the outer plexiform layer (OPL) serves as a synaptic site for photoreceptors, bipolar, and horizontal cells. The inner nuclear layer (INL) comprises signals from photoreceptors through bipolar, horizontal, and amacrine cells, with the inner plexiform layer (IPL) facilitating synaptic connections between bipolar, amacrine, and ganglion cells. The ganglion cell layer (GCL) transmits processed visual information to the brain through the axons of the ganglion cells, which form the RNFL, which make up the optic nerve (ON). Bruch’s membrane (BM) is the innermost layer of the choroid and is involved in regulation of fluid and solute passage to the retina [17] (Fig. 1).

The main objective of our study was to evaluate the effectiveness of CPAP in increasing the thickness of retinal layers. Other aims were to assess predictors of retinal and optic nerve damage in OSA and establish predictors of poor response to CPAP treatment in optic nerve damage.

To our knowledge, no previous studies have investigated HB and retinal and optic nerve damage in patients with OSA, and assessed the changes in the retina and optic nerve within one year of CPAP treatment.

## Methods

### Patient/subject groups

A prospective cohort study with consecutive inclusion of the first 3 patients who attended for treatment each day. All patients had previously undergone a sleep study at home or in hospital using a suitably validated respiratory polygraph device.

Patients diagnosed with moderate-severe OSA treated with CPAP (according to recommendations from the Spanish Society of Pneumology– SEPAR [18]) were recruited. The study lasted 2 years. OSA was diagnosed according to the AASM criteria [19] based on a manual reading of a polygraph from the Philips Respironics Alice PDx diagnostic recording device. Four weeks after the start of CPAP treatment, the equipment was titrated by downloading the Built-in-software (BIS) of the equipment. We considered correct titration of the equipment to be as follows: residual AHI < 10 events/hour, with unintentional leak < 24 L/min (< 95 percentile [95th ]) with nasal mask and < 36 L/min (< 95th ) with full face mask, over 3 consecutive nights). OCT was performed within 3 days of the patient’s inclusion and 12 months after the start of CPAP treatment.

Demographic variables recorded were age, sex, body mass index (BMI), arterial hypertension (AH), type 2 diabetes mellitus (T2DM), dyslipidaemia, smoking status, and Epworth score. Polygraphic variables analysed were AHI, cumulative percentage of time spent with oxygen saturation < 90% (CT90), mean oxygen saturation (SpO2), ODI, nocturnal respiratory failure (NRF), defined as CT90 ≥ 30% or mean SpO2 ≤ 90%, and HB, which was considered to be high when it was greater than p50 of the study population (> 100%min/h).

The study protocol and the informed consent form were approved by our Ethics Committee (CEIM) (Internal Code 2017.236), and the study was performed in accordance with the principles of the Declaration of Helsinki. Informed consent forms were signed by all participants prior to examinations.

### Inclusion and exclusion criteria

Patients between 18 and 75 years of age diagnosed with moderate-severe OSA (AHI ≥ 15) based on a respiratory polygraph with indication for CPAP treatment were included consecutively.

All participants underwent a through ophthalmic examination on the day of OCT imaging, comprising the following eye assessments: best-corrected visual acuity (Snellen charts), anterior segment biomicroscopy, refraction, OCT measurements, axial length (AL) assessment, intraocular pressure (IOP) quantification with Goldmann applanation tonometer (GAT) and dilated fundus examination. Participants received no pupil dilation drops to avoid changes in choroidal thickness [20]. The refractive error was recorded using an auto refractometer Canon RK-F1. Axial length (AL) was measured using the Lenstar LS 900. Each individual was randomised to decide which eye was to be examined first.

Exclusion criteria included previous CPAP treatment, the presence of respiratory failure (defined as SpO2 < 90% or PaO2 < 60 mmHg) or home oxygen therapy, an unstable situation, uncontrolled or acute psychiatric illnesses, heart failure (NYHA grades III or IV), central apnea (> 50% of the register with central apneas), a refractive error > 6.0 or < -6.0 diopters (D) of spherical equivalent or 3.0 D of astigmatism, any history of ocular surgery, ocular disease such as central serous chorioretinopathy, pachychoroid spectrum, uveitis and related macular degeneration, best corrected visual acuity as poor as 20/40, IOP ≥ 18 mmHg, past history of elevated IOP, neuroretinal rim notching, or optic disc hemorrhages. Similarly, other exclusion criteria included clinically relevant opacities of the optic media and low-quality images due to unstable fixation, or severe cataracts. All acquired spectral domain-OCT data sets had a quality score(Q) > 25.

### Optical coherence tomography assessment

OCT measurements were taken using Spectralis OCT. The examinations included horizontal and vertical non-isotropic scans measuring 8741 μm, resulting in dimensions of 8741 × 8741 µm^2^.

Retinal thickness was measured using spectral-domain (SD) Spectralis SD-OCT based on the images obtained by the posterior pole analysis scan.

**Fig. 2:**
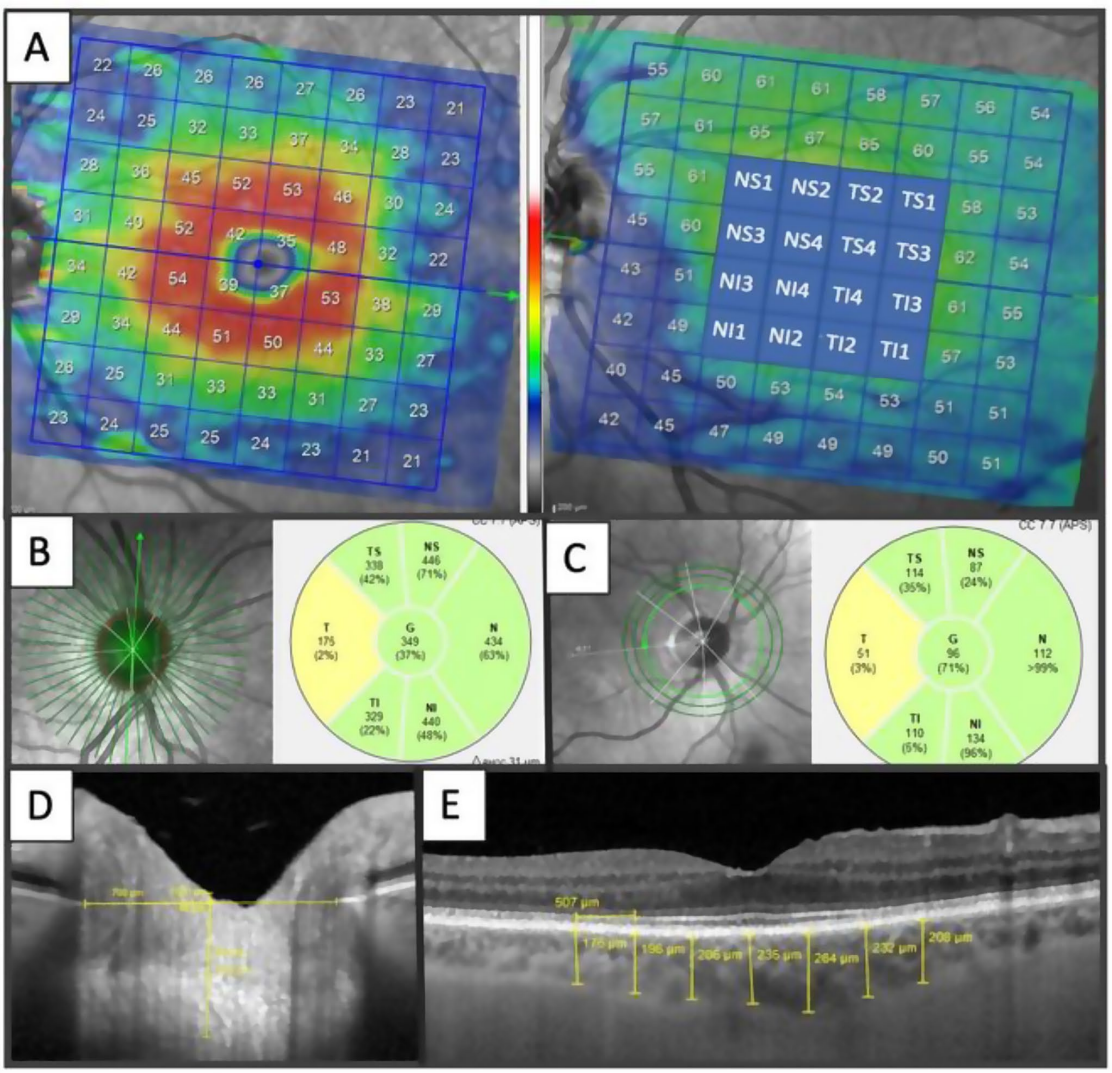
Optical coherence tomography parameters. **A**: Ganglion Cell Layer (GCL). **B**: Bruch’s membrane opening–minimum rim width (BMO-MRW). **C**: Retinal nerve fiber layer (RNFL), **D**: Lamina cribrosa (LC), **E**: Choroidal thickness (CT)

The average retinal layer measurement of each 8 × 8 (3˚x3˚) sector (64 sectors) was determined. Since glaucoma initially damages the centre of the macula, to determine the correlation between the ganglion cell-inner plexiform layer thickness measured with cirrus HD-OCT and macular visual field sensitivity measured with microperimetry [21], only 4 × 4 central grids were analysed to expedite the study (Fig. 2A). These 16 sectors were numbered as previously published [22], with temporal (T), nasal (N), superior (S) and inferior (I) added to help with understanding.

Bruch’s membrane opening-minimum rim width (BMO-MRW) is automatically centered at the optic nerve head, and 24 radial B-scans were acquired over a 15º area. The shortest distance from each identified BMO point to the internal limiting membrane (Fig. 2B) was measured.

RNFL thickness measurements of each individual eye were normalised for anatomic orientation of the fovea to optic nerve to an accurate and consistent positioning of the RNFL thickness measurement across eyes (automatic real-time tracking mean 100). Although the module includes 3 circle scans (inner circle: 3.5 mm, middle circle: 4.1 mm, and outer circle: 4.7 mm), we only recorded the figures provided by the inner circle scan (standard) (Fig. 2C). Six sector areas (superotemporal, superior, superonasal, inferonasal, inferior, and inferotemporal) and the average were measured in both analyses.

LC was measured by performing one vertical scan closest to the centre of the optic nerve head, at the point where the visibility of the anterior LC surface was as complete as possible, by excluding the main vessels using enhanced depth image technology, with an average of over 100 scans using the automatic averaging mode. A reference line connecting the two Bruch’s membrane end points was drawn, and one equidistant point (middle), with these then matched to the anterior prelaminar tissue surface and anterior LC surface (Fig. 2D).

Prelaminar tissue thickness (PTT) and anterior LC surface depth were measured at the three aforementioned points. Measurements were taken using the Spectralis software manual caliper tool by the aforementioned masked investigators (AC, AL).

### Statistical analysis

Accepting an alpha risk of 0.05 and a statistical power greater than 0.8 in a bilateral contrast, at least 23 subjects (46 observations) are required to detect a statistically significant difference equal to or greater than 5 units. A standard deviation for the first measurement is estimated at 11.38, and 6.89 for the second measurement (assuming a correlation of 0.2). A loss to follow-up rate of 0% is estimated. The sample size was calculated using Granmo v8.0.

A 1–sample Kolmogorov–Smirnov test was used to verify the normality of data distribution. A logistic regression analysis was performed considering the dependent variable of Bruch’s membrane opening-minimum rim width (BMO-MRW), RNFL and the independent variables as age, gender, BMI and cardiovascular risk factors (CVRF), AHI, CT90, mean Sp02, ODI and HB. For the selection of variables for the multivariate analysis, a univariate analysis was carried out with each of the variables according to the model proposed by Hosmer and Lemeshow [23]. Variables with a significance of less than 0.25 were considered, as well as their clinical relevance, regardless of their statistical significance.

All statistical analyses were performed using IBM SPSS Statistics V.20.0. The level of statistical significance was set at a p-value below 0.05.

## Results

Twenty-two patients (59.4%) were male and 15 (40.6%) were female. Seventy-two eyes from 37 OSA patients were enrolled in the study. All eyes included were phakic. Two eyes from two different patients were excluded due to amblyopia and a refractive error less than − 6 DP. The mean age and BMI were 59 ± 8 years and 34.39 ± 6.1 kg/m^2^, respectively. 25% of patients were diabetic, 61% hypertensive, and 44.4% dyslipidemic. In terms of smoking habits, 63.9% of patients had never smoked, 11.1% were active smokers and 25% ex-smokers. Regarding the polygraphic variables, the mean AHI was 46.30 ± 19.08, the mean CT90% was 21.4 ± 21.9%, the mean Sat02 was 91.2 ± 3% and the mean HB was 138. 16 ± 139.7%min/h (range: 13.6-588.5%min/h). The mean Epworth scale was 9.83 ± 3.94.

### Effectiveness of cpap treatment for retinal and optical nerve thickness

When comparing the measurements before starting CPAP therapy with those at 12 months, no improvement was observed in any of the lamina cribrosa (LC) and GCL measurements. In terms of RNFL, we did not observe an improvement in overall RNFL thickness, but we observed an improvement in the thickness of nasal RNFL (81.84 to 84 μm), inferonasal RNFL (111.24 to 113.08 μm) and superonasal RNFL (114.43 to 121.58 μm) without statistical significance. The BMO-MRW analysis revealed a significant increase in superotemporal BMO-MRW thickness after 12 months of CPAP treatment (316.54 to 318.23 μm, p-value = 0.008) (Table 1).

**Table 1:** Comparison of thickness of retinal nerve fibre layer (RNFL) and Bruch’s membrane opening-minimum rim width (BMO-MRW) 12 months after the start of CPAP treatment. SD: standard deviation. Avg: average, TS: superotemporal, T: Temporal, TI: inferotemporal, NI: inferonasal, N: nasal, NS: superonasal

	Before CPAP Mean ± SD	12 months Mean ± SD	*p*-value
RNFL avg (µm)	98.57 (8.37)	96.94 (11.43)	0.620
RNFL TS (µm)	129.89 (21.35)	125.17 (16.51)	0.903
RNFL T (µm)	69.38 (10.32)	63.42 (5.11)	0.314
RNFL TI (µm)	147.54 (17.81)	142.08 (13.15)	0.513
RNFL NI (µm)	111.24 (26.93)	113.08 (24.22)	0.578
RNFL N (µm)	81.84 (13.36)	84.00 (20.23)	0.076
RNFL NS (µm)	114.43 (19.63)	121.58 (20.24)	0.820
BMO-MRW avg (µm)	335.63(64.17)	332.87 (71.47)	0.981
BMO-MRW TS (µm)	316.54 (59.48)	318.23 (62.94)	**0.008**
BMO-MRW T (µm)	242.20 (52.15)	237.55 (50.35)	0.807
BMO-MRW TI (µm)	349.69 (73.06)	344.16 (82.60)	0.954
BMO-MRW NI (µm)	405.74 (91.99)	402.77 (106.45)	0.815
BMO-MRW N (µm)	370.11 (78.46)	370.06 (93.58)	0.332
BMO-MRW NS (µm)	385.74 (79.05)	379.26 (81.81)	0.819

### Assessment of retinal damage predictors

Using BMO-MRW as the dependent variable in logistic regression (Table 2), age, sex, T2DM and HB were significant in the univariate analysis. When a multivariate analysis was performed, it was determined that increased HB (> 100%min/h) was the variable associated with a significantly increased odds ratio (OR) of retinal and optic nerve damage (OR = 3.58, *p* = 0.03), with this being a predictor of retinal and optic nerve damage.

**Table 2:** Logistic regression analysis for assessment of predictors of retinal damage considering BMO-MRW as the dependent variable. OR: odds ratio. BMI: body mass index, AHI: Apnea-Hypopnea index, CT90: cumulative percentage of time spent with oxygen saturation < 90%, NRF: nocturnal respiratory failure, ODI: oxygen desaturation index, T2DM: type 2 diabetes mellitus, AH: arterial hypertension

Univariate Analysis (BMO-MRW)		
Variable	Or	*p*-value
Sex (male)	2.09	**0.14**
Age	0.97	**0.22**
BMI	0.95	0.26
AHI	1.00	0.79
Mean Sp02	1.07	0.39
CT90	0.99	0.36
NRF	0.79	0.63
ODI	1.01	0.55
T2DM	2.55	**0.10**
AH	0.77	0.61
Dyslipidemia	1.27	0.63
Hypoxic Burden	2.44	**0.08**
Multivariate Analysis (BMO-MRW)		
Variable	**OR**	**p-value**
Sex (male)	2.93	0.89
Age	0.43	0.85
T2DM	3.19	0.89
Hypoxic Burden	3.58	**0.03**

In a univariate analysis taking RNFL as a dependent variable (Table 3), age, respiratory insufficiency, T2DM, AHT and HB were significant. When a multivariate analysis was performed, the T2DM was found to be a predictor of retinal and optic nerve damage (OR = 4.344, *p* = 0.042).

**Table 3:** Logistic regression analysis for assessment of predictors of retinal damage considering RNFL as the dependent variable. OR: odds ratio. BMI: body mass index, AHI: Apnea-Hypopnea index, CT90: cumulative percentage of time spent with oxygen saturation < 90%, NRF: nocturnal respiratory failure, ODI: oxygen desaturation index, T2DM: type 2 diabetes mellitus, AH: arterial hypertension

Univariate Analysis (RNFL)		
Variable	Or	*p*-value
Sex (male)	1.00	1.00
Age	1.08	**0.02**
BMI	0.97	0.55
AHI	0.99	0.48
Mean Sp02	0.93	0.39
CT90	1.01	0.40
NRF	2.65	**0.05**
ODI	0.99	0.38
T2DM	3.59	**0.03**
AH	5.25	**0.01**
Dyslipidemia	1.00	1.00
Hypoxic Burden	0.53	**0.21**
Multivariate Analysis (RNFL)		
Variable	**OR**	**p-value**
Sex (man)	0.33	0.15
Age	1.16	0.14
NRF	2.79	0.14
T2DM	4.34	**0.04**
AH	3.04	0.13
Hypoxic Burden	0.32	0.10

### Evaluating the predictors of poor response to cpap treatment

Logistic regression analysis was performed using differences in BMO-MRW thickness after 12 months of CPAP treatment as a dependent variable (Table 4). An univariate analysis demonstrated that age, BMI, NRF, AH were significant. When a multivariate analysis was performed, none of the variables presented a significantly high OR.

**Table 4:** Logistic regression analysis for assesament of predictors of poor response to CPAP treatment considering the difference in BMO-MRW thickness 12 months after CPAP treatment as the dependent variable. OR: odds ratio. BMI: body mass index, AHI: Apnea-Hypopnea index, CT90: cumulative percentage of time spent with oxygen saturation < 90%, NRF: nocturnal respiratory failure, ODI: oxygen desaturation index, T2DM: type 2 diabetes mellitus, AH: arterial hypertension

Univariate Analysis (BMO-MRW 0–12 m)		
Variable	Or	*p*-value
Sex (male)	0.95	0.92
Age	0.96	**0.18**
BMI	1.15	**0.01**
AHI	1.12	0.29
Mean Sp02	1.00	0.99
CT90	1.01	0.35
NRF	2.24	**0.1**
ODI	1.01	0.33
T2DM	1.43	0.53
AH	0.42	**0.12**
Dyslipidemia	2.62	**0.07**
Hypoxic Burden	1.20	0.73
Multivariate Analysis (BMO-MRW 0–12 m)		
Variable	**OR**	**p-value**
Sex (male)	0,77	0.71
Age	0.96	0.40
BMI	1.13	0.06
NRF	1.75	0.46
AH	0.73	0.73
Dyslipidemia	0.33	0.08

Regarding the difference in RNFL thickness (Table 5), in the univariate analysis none of the variables were significant.

**Table 5:** Logistic regression analysis for assessment of predictors of poor response to CPAP treatment considering the difference in RNFL thickness 12 months after treatment with CPAP as the dependent variable. OR: odds ratio. BMI: body mass index, AHI: Apnea-Hypopnea index, CT90: cumulative percentage of time spent with oxygen saturation < 90%, NRF: nocturnal respiratory failure, ODI: oxygen desaturation index, T2DM: type 2 diabetes mellitus, AH: arterial hypertension

Univariate Analysis (RNFL 0–12 m)		
Variable	Or	*p*-value
Sex (male)	0.79	0.33
Age	1.02	0.48
BMI	0.97	0.50
AHI	1.00	0.99
Mean Sp02	1.01	0.87
CT90	1.00	0.73
NRF	0.72	0.50
ODI	1.00	0.70
T2DM	1.02	0.97
AH	1.30	0.60
Dyslipidemia	1.05	0.92
Hypoxic Burden	1.12	0.83

## Discussion

OSA is associated with ophthalmologic disease due to hypoxia, proinflammatory state, production or reactive oxygen species, and intrathoracic pressure changes [14]. Furthermore, perfusion changes increase vessel resistance and blood viscosity which can cause ischaemia. It is known that in moderate and severe OSA, the inner diameter of the ophthalmic artery decreases [24].

Despite a close link between OSA and neuro-ophthalmological diseases, there are no studies linking HB with retinal and optic nerve damage. In our case, we observed that patients with high HB (> 100%min/h) were 3.6-times more likely to suffer a superotemporal BMO-MRW thinning, which indicates that HB may be a predictor of retinal and optic nerve damage. It is the first time that this result has been reported.

OCT is a non-invasive method used to evaluate neuronal and axonal changes, notably unmyelinated intraocular nerve fibres, and can detect retinal damage in its early stages. New generation SD-OCT segmentation algorithms such as BMO-MRW, have been developed to improve optic nerve analysis [25]. Many studies found that BMO-MRW is a more sensitive and specific parameter than RNFL to evaluate damage to the optic disc [25, 26]. Previous studies measured RNFL thickness as a marker of retinal damage and disease severity showing a significant correlation [27]. Nevertheless, changes in BMO-MRW develop earlier than RNFL and can precede vision loss [28].

In our study, we found that BMO-MRW detect CPAP changes more accurately than RNFL, so we believe it should be the parameter used to assess retinal response to CPAP in OSA. Our study is also the first to assess CPAP-induced changes after one year of treatment. There has only been one study that has assessed changes at 6 months after treatment and this measured RNF [8]. In this study, the patients had severe OSA with mean AHI of 55.4 ± 21.12/h, which is a limitation in the application of results as it does not represent usual clinical practice in the population. In our case, we assessed patients with moderate and severe OSA with mean AHI of 46.30 ± 19.08 which is a strength of the work.

CPAP therapy reduces upper airway collapse which improves hypoxia, reduces oxidative stress, and increases optic nerve perfusion [29]. However, its long-term benefit has not been determined by OCT. In our case, CPAP produced a significant improvement in superotemporal BMO-MRW thinning indicating this long-term retinoprotective and neuroprotective role in areas with reversible damage. This increase in thickness agrees with the study by Batum et al. [8], in which they reported a significant thickening of several sectors of the RNFL and the foveal macular layer 6 months after the start of CPAP treatment in patients with severe OSA. In terms of predictors of CPAP response, we did not find any related variables.

In our opinion, HB could be used to detect patients with OSA and a higher risk of retinal damage. Usually, diagnosis of neuro-ophthalmological diseases takes place after the onset of symptoms when there is already established, sometimes irreversible damage [29].

We propose that OCT should be performed in patients with OSA and neuro-ophthalmological symptoms or asymptomatic patients, especially in patients with T2DM and elevated HB.

This study has several limitations. Firstly, we have not analysed subgroups of patients with moderate or severe OSA. However, CPAP is currently considered the standard treatment in patients with moderate or severe OSA. This finding is a strength of the results as it reflects the population treated in daily practice. Secondly, we only used the central 16 sectors of the GCL. However, publications have demonstrated that these sectors are very useful for detecting glaucomatous damage [30]. Other limitations are the relatively small sample size and the fact that the sample was ethnically homogeneous.

In conclusion, BMO-MRW thickness can provide an earlier assessment of retinal involvement in patients with OSA and may allow assessment of the impact of CPAP treatment. HB is a predictor of retinal and optic nerve damage in patients with OSA. CPAP treatment may have a long-term protective effect on the retina and optic nerve increasing the thinning of the areas affected by the disease. Early detection of patients with OSA at high risk of retinopathy is necessary to avoid irreversible damage.

## Data Availability

The datasets generated during and/or analyzed during the current study are available from the corresponding author on reasonable request.

## Declarations

### Artificial intelligence

In this article there is not an involvement of artificial intelligence.
